# Accurate characterization of ^99m^Tc-MDP uptake in extraosseous neoplasm mimicking bone metastasis on whole-body bone scan: contribution of SPECT/CT

**DOI:** 10.1186/s12880-019-0345-1

**Published:** 2019-05-30

**Authors:** Linqi Zhang, Qiao He, Tao Zhou, Bing Zhang, Wei Li, Hao Peng, Xi Zhong, Liwu Ma, Rusen Zhang

**Affiliations:** 1 Department of Nuclear Medicine, Affiliated Cancer Hospital & Institute of Guangzhou Medical University, 78 Hengzhigang Road, Guangzhou, 510095 Guangdong province People’s Republic of China; 2 Department of Nuclear Medicine, the First Affiliated Hospital of Sun Yat-Sen University, 58 Zhongshan Er Road, Guangzhou, 510080 Guangdong province People’s Republic of China; 3 Department of Radiology, Affiliated Cancer Hospital & Institute of Guangzhou Medical University, 78 Hengzhigang Road, Guangzhou, 510095 Guangdong province People’s Republic of China

**Keywords:** Extraosseous uptake, ^99m^Tc-MDP, SPECT/CT, Whole-body bone scan, Calcification

## Abstract

**Background:**

^99m^Tc-labelled methylene diphosphonate (^99m^Tc-MDP) uptake can occasionally be identified in extraosseous neoplasms on whole-body scans (WBSs) and may be misinterpreted as bone metastasis. The purpose of our study was to investigate the frequency of ^99m^Tc-MDP uptake in extraosseous neoplasms and to assess the additional value of SPECT/CT for the localization and characterization of this unusual uptake.

**Methods:**

Data from 7308 patients (SPECT/CT was performed in 2147 patients) with known cancer who underwent WBSs for metastatic work-up between May 2015 and July 2018 were retrospectively reviewed. The locations, numbers, and intensities of extraosseous ^99m^Tc-MDP uptake were evaluated by WBS, and the intratumoural calcification was evaluated by SPECT/CT. The diagnostic accuracy of SPECT/CT in locating ^99m^Tc-MDP uptake in extraosseous neoplasms was compared to that of WBS.

**Results:**

A total of 41 patients showed ^99m^Tc-MDP uptake in extraosseous neoplasms. Of these patients, 23 patients had uncertain lesions by WBS, and further SPECT/CT was performed. The incidence of ^99m^Tc-MDP uptake in extraosseous neoplasms was observed to be 0.6% by WBS and 1.1% (by) SPECT/CT. During imaging analysis, WBS had an accuracy of only 35% (14/40), whereas SPECT/CT correctly located and diagnosed all 40 lesion sites in the 23 patients. Twenty-three lesion sites (57.5%, 23/40) showed moderate or high intensity of extraosseous ^99m^Tc-MDP uptake. Of the 23 patients, 17 patients (73.9%, 18/23) with 31 lesion sites (77.5%, 31/40) presented with intratumoural calcification.

**Conclusions:**

^99m^Tc-MDP uptake in extraosseous neoplasms can be observed as 0.6% on WBS and is usually localized to the breast, liver, and lung. Nuclear physicians should be familiar with such extraosseous uptake when interpreting WBSs. SPECT/CT offers better accuracy than WBS alone for locating the majority of lesions present with intratumoural calcification.

## Background

Whole-body bone scans (WBSs) using ^99m^Tc-labelled methylene diphosphonate (^99m^Tc-MDP) is the most sensitive examination for the detection of bone metastasis in patients with known cancer [1, 2]. Normally, ^99m^Tc-MDP uptake is seen in the skeletal structure and urinary system. However, a few studies have suggested that ^99m^Tc-MDP uptake can occasionally be identified in some extraosseous neoplasms (including primary tumour [3–5] and metastatic lesions [6–8]) by WBS, which may be misinterpreted as bone metastases or skeletal involvement. To distinguish this finding from bone metastasis is important for patients with known cancer; however, this is particularly difficult if WBS is used alone because of limited anatomical resolution. SPECT/CT offers the opportunity to acquire both anatomical and functional images, hence enabling more precise anatomical localization and characterization of abnormal radiotracer uptake by SPECT using CT images, possibly reducing confusion and enhancing the diagnostic value of the study when interpreting WBS images question, thereby altering patient management. [1, 9–11].

In the last few decades, various authors have reported the potential value of SPECT/CT in the localization and characterization of ^99m^Tc-MDP uptake in extraosseous neoplasms, including breast cancer, gastrointestinal stromal tumour, Ewing sarcoma, pyelonephritis, sclerosing pneumocytoma, and other diseases [3–8, 12–15]. Generally, these case reports and pictorial essays refer to a single case or to a limited number of patients. Nevertheless, the incidence and imaging features of SPECT/CT in such extraosseous uptake have not been systematically summarized, and the exact mechanisms remain unclear. Therefore, the aims of present study were first, to investigate the frequency of extraosseous uptake of ^99m^Tc-MDP in soft-tissue neoplasms and to discuss the possible mechanism, and second, to assess the additional value of SPECT/CT in the localization and characterization of this unusual uptake compared to WBS alone.

## Methods

### Patients

Between May 2015 and July 2018, ^99m^Tc-MDP WBS was performed in 7308 patients (SPECT/CT was performed in 2147 patients) at our hospital. Patients were included in our study if they met the following criteria: (1) known cancer undergoing WBS for metastatic work-up; and (2) WBS or further SPECT/CT found to have ^99m^Tc-MDP uptake in extraosseous neoplasm. This study was approved by the local ethics committee. Because of the retrospective nature of the study, written informed consent was waived.

### Imaging acquisition

All images were acquired using a SPECT/CT scanner (Philips Healthcare, Eindhoven, the Netherlands). WBS images were acquired 2–3 h after intravenous injection of ^99m^Tc-MDP at 15–25 mCi, using a low energy, high resolution, and parallel hole collimators. A 256 × 256 matrix with 500 K counts per view was used. After WBS acquisition, the imaging was directly interpreted by experienced nuclear medicine physician. If uncertain lesions were identified on WBS, one or more additional SPECT/CT scans were performed. A low-dose CT was performed for anatomic location and attenuation correction. CT data were acquired with exposure of 140 KeV, 2.5 mA, and 512 × 512 matrix. After CT acquisition, the SPECT acquisition protocol was started, as follows: 15% energy window at 140 keV, and 128 × 128 matrix. Processing and fusion of SPECT and CT images were performed using Jet Steam Workspace (Philips Healthcare, Eindhoven, Netherlands).

### Imaging analysis

All images were reviewed by two nuclear medicine physicians with interpretation in consensus. In the first step, reviewers evaluated the WBS images while unaware of the SPECT/CT images, according to following imaging features: location, number, and intensity of extraosseous ^99m^Tc-MDP uptake. When interpretating WBS images, abnormal radiotracer uptake lesions were classified as two categories (extraosseous and intraosseous). The criteria for classifying a region of ^99m^Tc-MDP uptake as extraosseous were: the lesion was clearly outside the skeleton structure (such as in chest wall and abdominal region), and the region could not be accounted for by physiological or other distribution. The criterion for classifying a region of ^99m^Tc-MDP uptake as intraosseous was: the lesion overlapped with the skeletal structure. The intensity of ^99m^Tc-MDP uptake was graded as high (the ^99m^Tc-MDP uptake was higher than the sternum on the WBS images), moderate (equal to sternum), or low (less than the sternum). In the second step, SPECT/CT images were evaluated according to the following imaging features: location, number, and presence or absence of intratumoural calcification. For the definition of a disease site, we used the following criteria: (1) each involved liver segment, (2) each lymph node region, (3) each lung lobe and (4) every soft tissue neoplasm.

### Statistical analysis

Continuous data were described as the means±standard deviations (SD). Categorical data were described as numbers and frequency (%). The diagnostic accuracy of SPECT/CT for locating ^99m^Tc-MDP uptake in extraosseous neoplasm were calculated and compared to WBS using the chi-square test. *P* values less than 0.05 were considered statistically significant. All statistical data were performed using the SPSS Statistics 17.0 (SPSS Inc., Chicago, IL, USA) software.

## Results

### Patients

A total of 41 patients showed ^99m^Tc-MDP uptake in extraosseous neoplasms. Of these patients, 23 patients had uncertain lesions on WBS, and one or more additional SPECT/CT scans were performed. Patient characteristics are detailed in Table 1. The remaining 18 patients with breast cancer showed ^99m^Tc-MDP uptake in the anterior chest wall by WBS, suggesting extraosseous uptake in breast cancer, and further SPECT/CT was not performed. The incidence of ^99m^Tc-MDP uptake in extraosseous neoplasms was 0.6% (41/7308) by WBS and 1.1% (23/2147) by SPECT/CT.

**Table 1:** Characteristics of 23 patients with radiopharmaceutical uptake in extraosseous neoplasms

Characteristics	Value	Percentage (range)
Age (years)	57.4 ± 12.1	33–78
Sex		
Male	12	52.2%
Female	11	47.8%
History of malignancy		
Breast cancer	7	30.4%
Lung cancer	4	17.4%
Colorectal cancer	3	13.0%
Ovarian tumor	2	8.7%
Osteosarcoma	2	8.7%
Gastric stromal tumor	1	4.3%
Endometrial cancer	1	4.3%
Thyroid cancer	1	4.3%
Malignant teratoma	1	4.3%
Hepatocellular cancinoma	1	4.3%
Primary treatment		
No treatment	16	69.6%
Surgery + chemotherapy +RT	3	13.0%
Surgery + chemotherapy	4	17.4%

*RT* radiotherapy

**Fig. 1:**
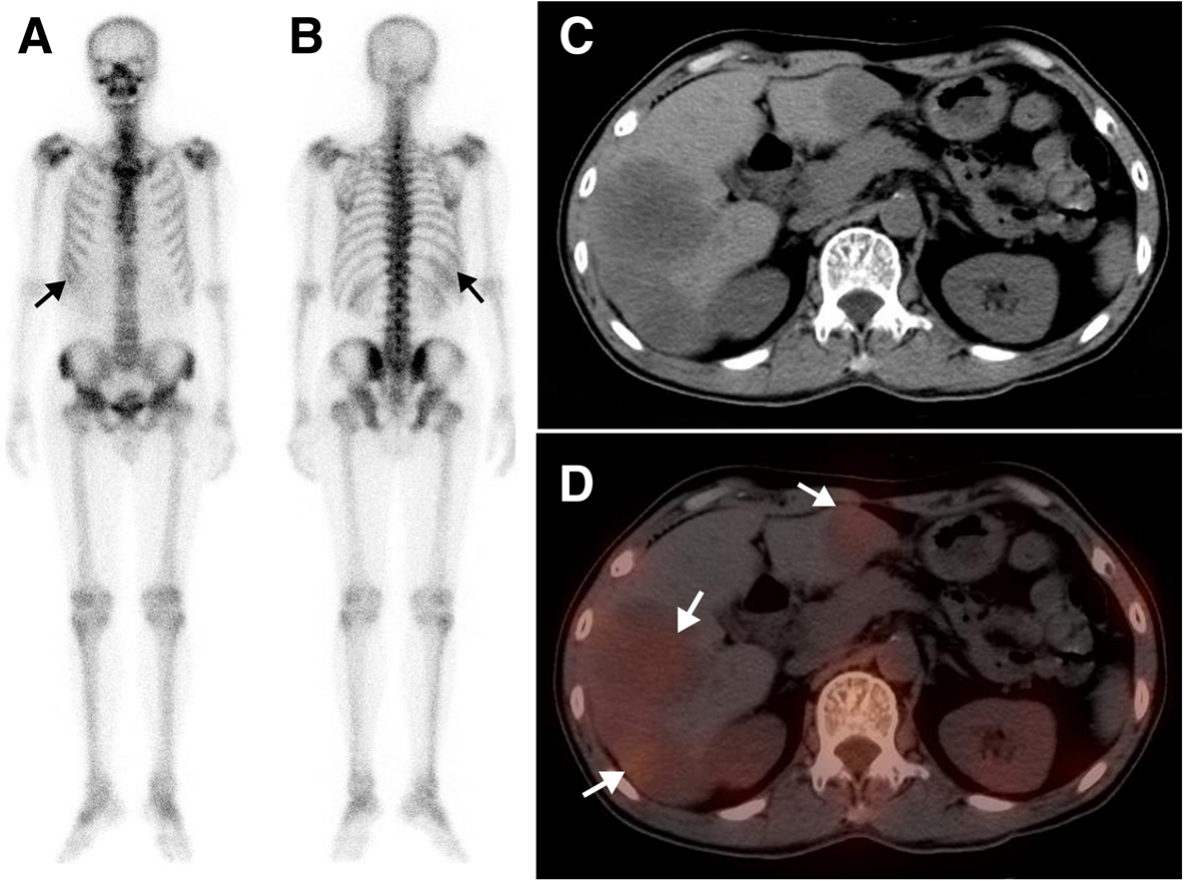
A 62-year-old man with rectal cancer after surgery and chemotherapy for 3 years, who was referred for suspected metastasis. The whole-body scan (**a**, anterior; **b**, posterior) demonstrated ^99m^Tc-MDP uptake in right upper abdomen (black arrow). Axial CT (**c**), and SPECT/CT images (**d**) showed increasing ^99m^Tc-MDP uptake corresponding to multiple liver metastases without calcification (white arrow)

**Fig. 2:**
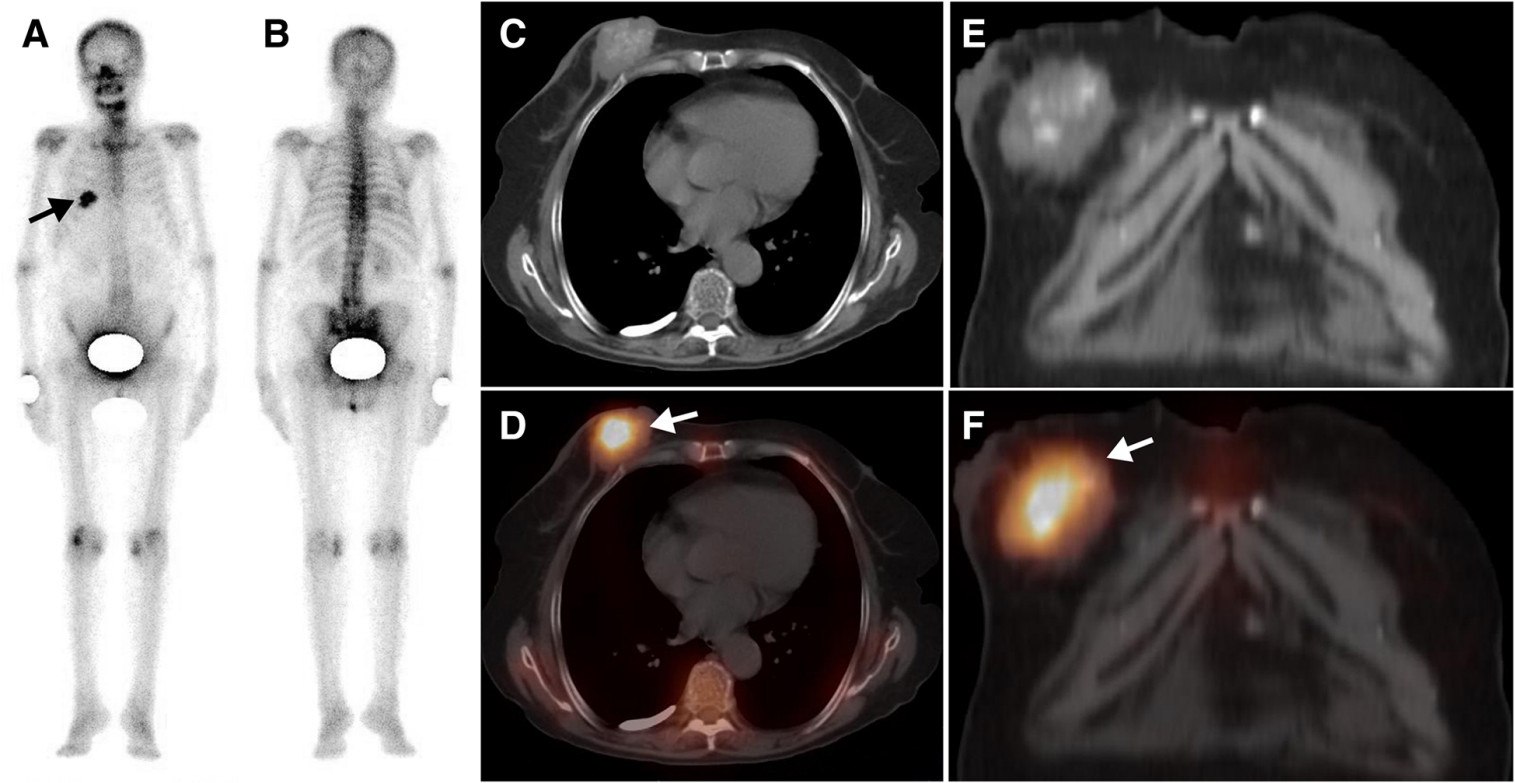
A 76-year-old woman with breast cancer was referred for metastatic work-up before surgery. The whole-body scan (**a**, anterior; **b**, posterior) revealed a focal area of high ^99m^Tc-MDP activity in the right 5-6th posterior rib region (black arrow). Axial CT (**c**), and coronal CT (**e**) and SPECT/ CT images (**d**, **f**) showed that elevated ^99m^Tc-MDP activity was located in a breast tumour with calcification (white arrow)

**Fig. 3:**
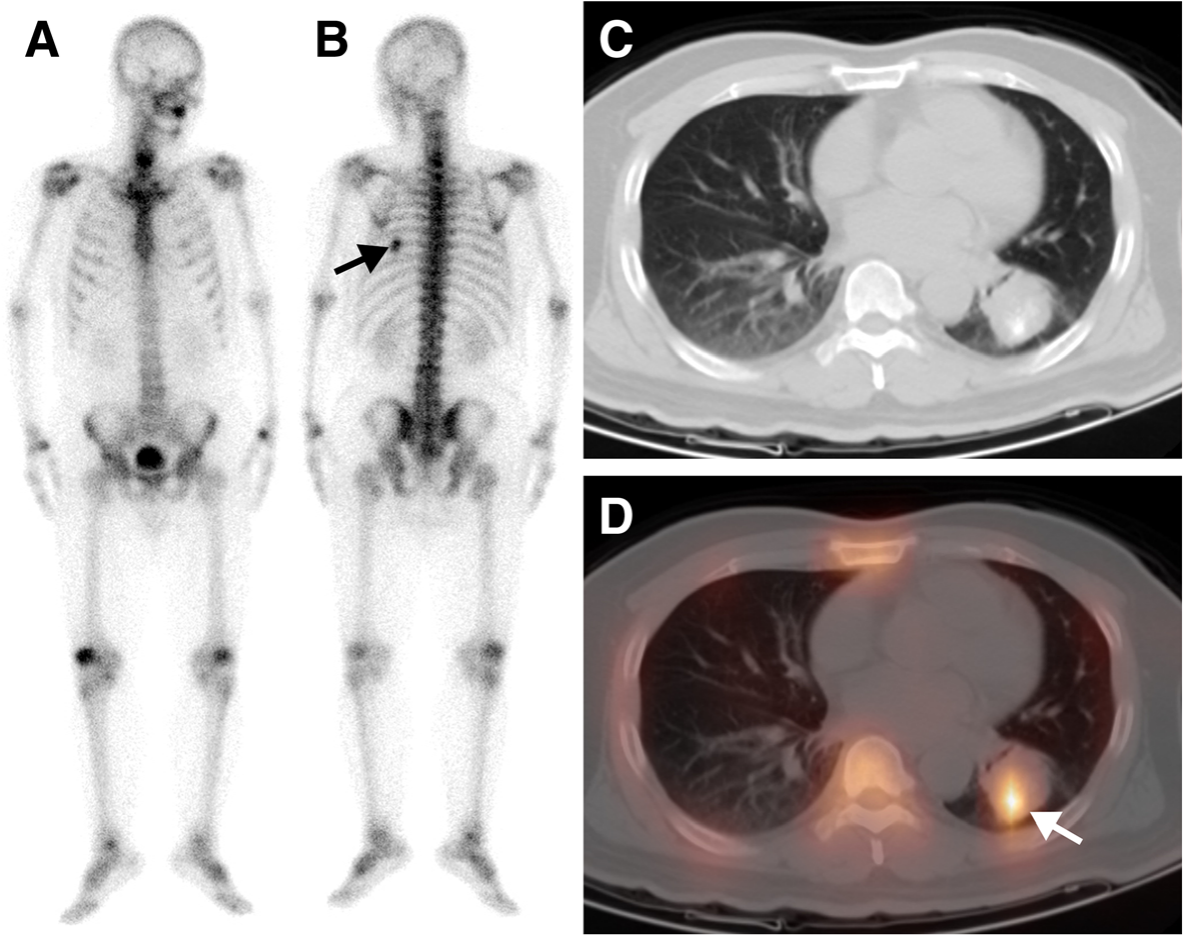
A 66-year-old man with a known gastric stromal tumour was referred for metastatic work-up before surgery. The whole-body scan (**a**, anterior; **b**, posterior) revealed a single focus of high ^99m^Tc-MDP activity in the right 7th posterior rib region (black arrow). Axial CT (**c**), and SPECT/ CT images (**d**) showed that the elevated ^99m^Tc-MDP activity was located in a pulmonary mass with calcification (white arrow). The patient underwent a left lower lobectomy and pathologic analysis confirmed the diagnosis of a lung adenocarcinoma

**Fig. 4:**
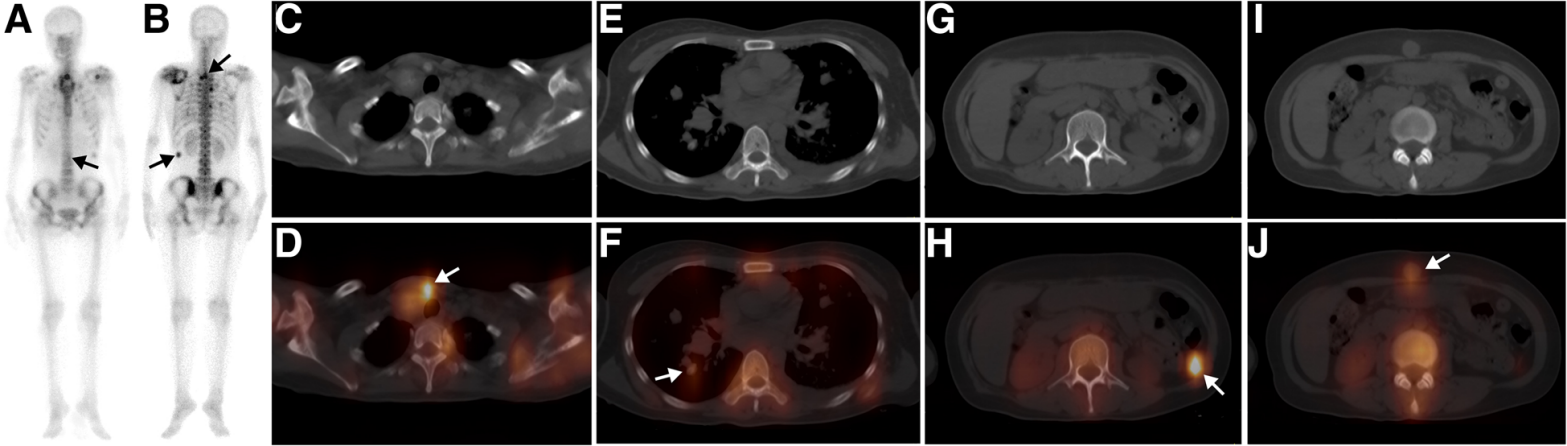
A 59-year-old man with known scapular osteosarcoma was referred for metastatic work-up. The whole-body scan (**a**, anterior; **b**, posterior) showed multiple foci of abnormally increased ^99m^Tc-MDP uptake in the left shoulder, supraclavicular region, chest and abdomen (black arrow). Axial CT (**c**, **e**, **g**, **i**), and SPECT/ CT images (**d**, **f**, **h**, **j**) showed that the elevated ^99m^Tc-MDP activity was located in the left shoulder, supraclavicular lymph nodes, pleura, lung, posterior peritoneum and periumbilical subcutaneous tissue (the lesion was missed on WBS due to overlap with the lumbar vertebra) (white arrow)

**Fig. 5:**
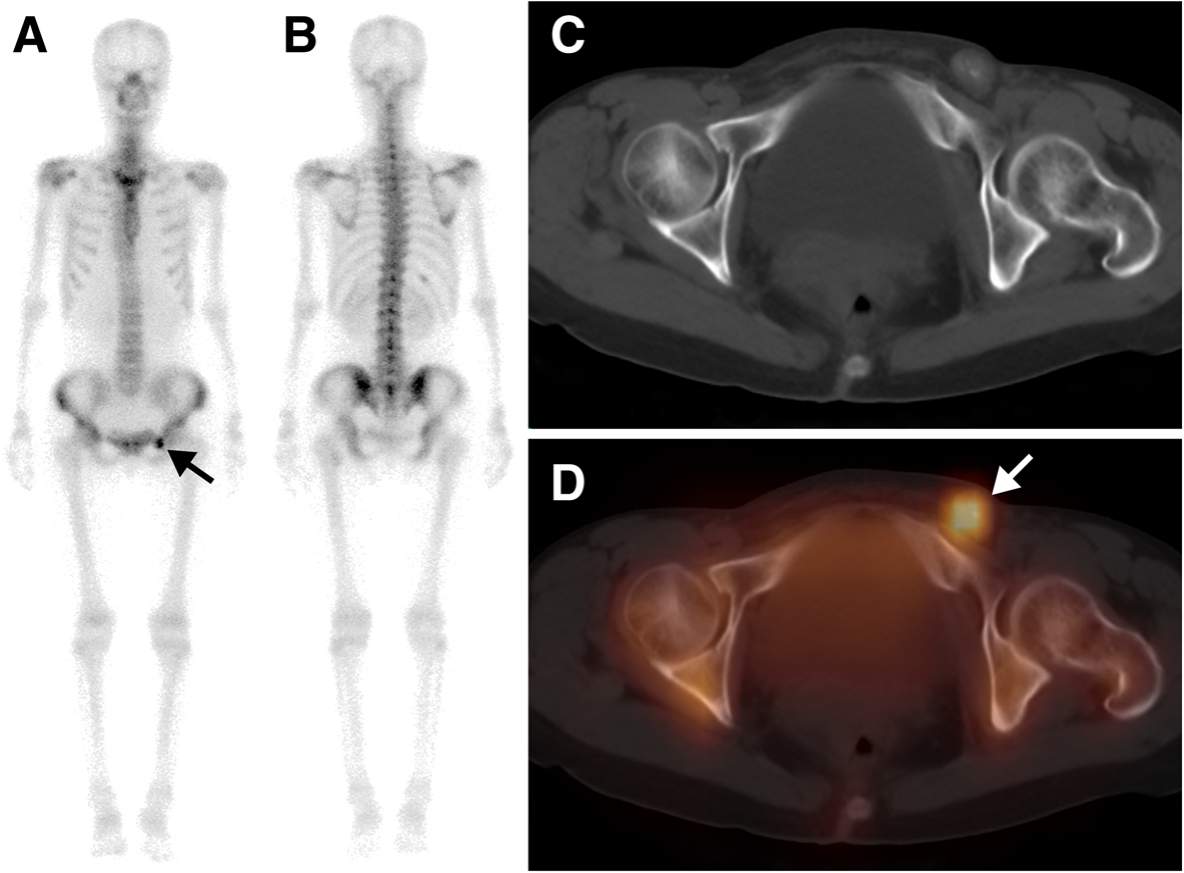
A 40-year-old woman with ovarian cancer (papillary adenocarcinoma) after surgery and chemotherapy for 2 years, who was referred for routine follow-up. The whole-body scan (**a**, anterior; **b**, posterior) revealed a single focus of high ^99m^Tc-MDP activity in the left pubic bone region (black arrow). Axial CT (**c**), and SPECT/ CT images (**d**) showed that the elevated ^99m^Tc-MDP activity was located in a left inguinal lymph node with calcification (white arrow). Resection of the lymph node was performed and pathologic analysis confirmed the diagnosis of lymph node metastases

**Fig. 6:**
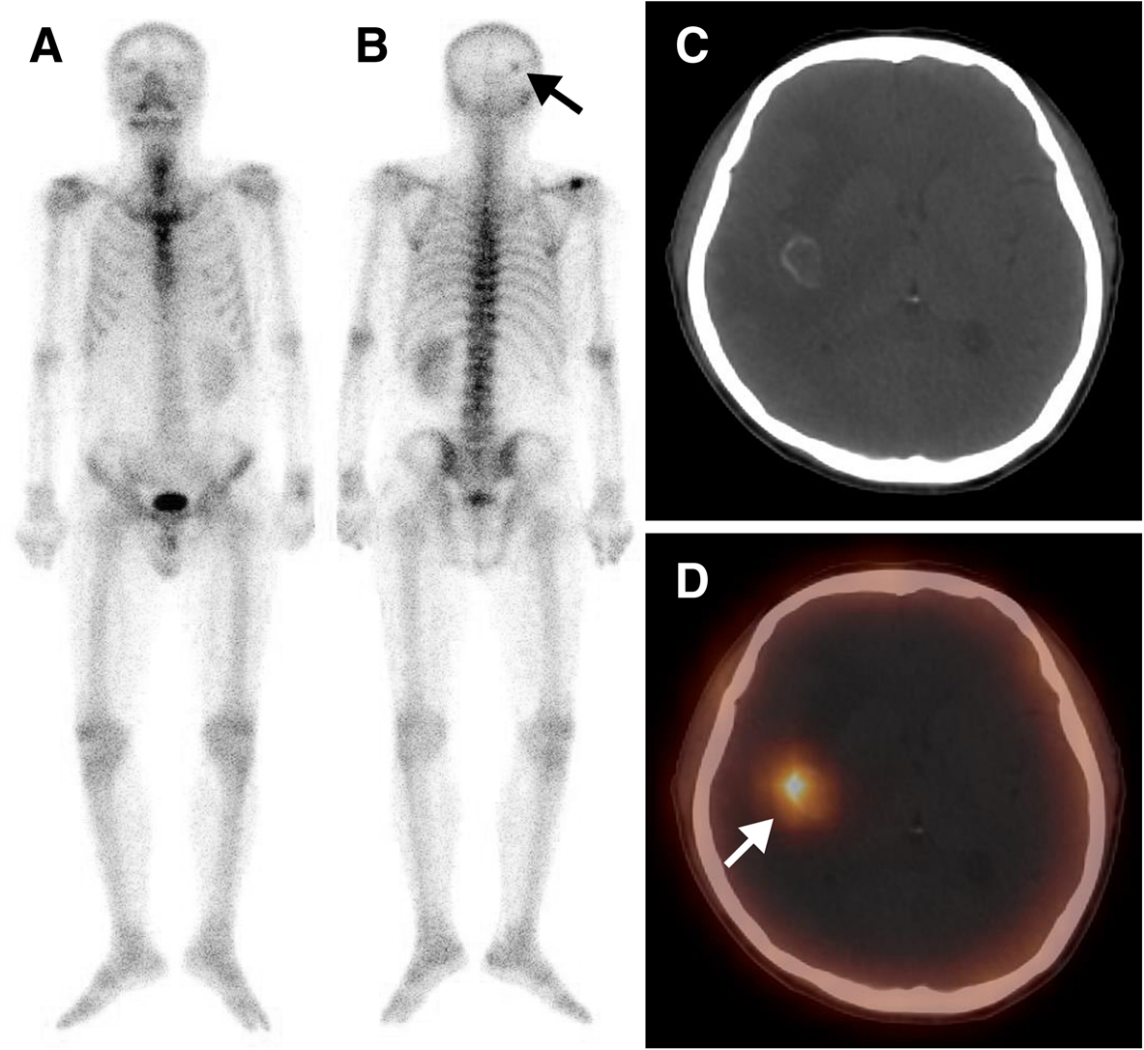
A 49-year-old man with lung cancer (adenocarcinoma) after surgery and chemotherapy for 1 year, who was referred for suspected metastasis. The whole-body scan (**a**, anterior; **b**, posterior) revealed a single focus of high ^99m^Tc-MDP activity in the right occipital bone (black arrow). Axial CT (**c**), and SPECT/ CT images (**d**) showed elevated ^99m^Tc-MDP activity in a brain metastasis at the grey-white matter junction in the right parietal lobe (white arrow)

In total, SPECT/CT detected 40 sites of extraosseous ^99m^Tc-MDP uptake in the 23 patients (Table 2). ^99m^Tc-MDP uptake in extraosseous neoplasms were most frequently found in liver (Fig. 1), accounting for 30% (12/40) of lesions, followed by breast (Fig. 2), lung (Figs. 3 and 4), pleura, lymph node (Figs. 3 and 5), peritoneum (Fig. 4), subcutaneous soft tissues (Fig. 4), ovary, brain (Fig. 6), chest wall, thyroid, mediastinum, and uterus.

**Table 2:** Anatomical location and final diagnosis of 40 sites of radiopharmaceutical uptake in extraosseous neoplasms found on SPECT/CT

Lesion sites	Primary	Metastasis	Total	Percentage
Liver	1	11	12	30%
Breast	6	0	6	15%
Lung	2	2	5	12.5%
Pleura	0	3	3	7.5%
Lymph node	0	3	3	7.5%
Peritoneum	0	2	2	5%
Subcutaneous soft tissues	0	2	2	5%
Ovary	2	0	2	5%
Chest wall	0	1	1	2.5%
Brain	0	1	1	2.5%
Uterus	1	0	1	2.5%
thyroid	1	0	1	2.5%
Mediastinum	1	0	1	2.5%
Total	14	26	40	100%

### WBS and SPECT/CT findings

A summary of the clinical characteristics, WBS and SPECT/CT findings of all 23 patients is displayed in Table 3. On the WBS images, the intensity of extraosseous ^99m^Tc-MDP uptake was considered high in 17 lesion sites, moderate in 6 sites and low in 17 sites. Fourteen lesion sites showing abnormal areas of ^99m^Tc-MDP uptake outside the skeleton structure were interpreted as having extraosseous uptake. Twenty-three sites of lesion overlapping with the skeleton structure were misinterpreted as having bone lesions. Three sites of lesion were missed by WBS because of extraosseous ^99m^Tc-MDP uptake equal to or lower than that of adjacent bone. Overall, when considering extraosseous uptake lesions as positive, WBS had an accuracy of only 35% (14/40) based on the standard criteria.

**Table 3:** Clinical data and SPECT/CT findings of radiopharmaceutical uptake in extraosseous neoplasms in 23 patients

Pat.	Clinical diagnosis	Primary treatment	WBS		SPECT/CT finding		Final diagnosis
			Localization	Intensity (n)	Localization	CA	
1	Gastric stromal tumor	NA	L. 7th posterior rib	H (1)	L. Lung	+	Lung cancer (adenocarcinoma)
2	Lung cancer	NA	R. Anterior chest wall	L (1)	R. Lung	+	Lung cancer (adenocarcinoma)
3	Lung cancer	Surgery + chemotherapy +RT	R. occipital bone	H (1)	R. Brain	+	Brain metastasis
4	Breast cancer	NA	R. Anterior chest wall	M (1)	R. Breast	+	Breast cancer
5	Breast cancer	NA	R. 3th posterior rib	M (1)	R. Breast	_	Breast cancer
6	Breast cancer	NA	R. 5-6th posterior rib	H (1)	R. Breast	+	Breast cancer
7	Breast cancer	NA	R. Anterior chest wall	L (1)	R breast	+	Breast cancer
8	Breast cancer	Surgery + chemotherapy +RT	R.6th posterior rib	H (1)	R. anterior chest wall	_	Recurrent breast cancer
9	Breast cancer	NA	L. Anterior chest wall	L (1)	L breast	_	Breast cancer
10	Breast cancer	NA	R. Anterior chest wall	H (1)	R breast	+	Breast cancer
11	Rectal cancer	Surgery + chemotherapy	R. 4-5th posterior rib	M (1)	R. Lung	+	Lung metastasis
12	Colorectal cancer	Surgery + chemotherapy	R. Upper abdomen	L (4)	Liver	+	Liver metastasis
13	Rectal cancer	Surgery + chemotherapy	R. Upper abdomen	L (4)	Liver	+	Liver metastasis
14	Ovarian cancer	Surgery + chemotherapy	L. Pubic bone	H (1)	Lymph node	+	Lymph node metastasis
15	Ovarian cancer	NA	Abdominopelvic cavity	H (1)	Peritoneum	+	Peritoneal metastasis
16	Breast cancer	NA	L. Pelvic cavity	H (1)	Ovary	+	Ovarian thecoma
17	Osteosarcoma	NA	L. Supraclavicular region Multiple rib L. Upper abdomen Third lumbar vertebra	H (4) M (2) L (2)	L. Supraclavicular Lymph node R. Lung L. Upper peritoneum Subcutaneous soft tissues	+	Multiple metastasis
18	Osteosarcoma	NA	Multiple rib	H (2)	Mediastinal lymph node	+	Lymph node metastasis
19	Primary liver malignancy	NA	R. Upper abdomen	H (1)	Liver	+	HCC
20	Thyroid cancer	NA	Lower cervical vertebra	M (1)	Right thyroid	–	Medullary thyroid cancer
21	Uterine malignancy	NA	R. Sacroiliac joint	H (1)	Uterus	–	Endometrial cancer
22	Mediastinum tumor	NA	R. Anterior chest wall	H (1)	Mediastinum	+	Malignant teratoma
23	Lung cancer	Surgery + chemotherapy +RT	R. Upper abdomen	L (3)	Liver	_	Liver metastasis

*RT* radiotherapy, *L* left, *R* right, *H* high, *M* moderate, *L* low, *CA* calcification, *HCC* hepatocellular carcinoma

With the addition of SPECT/CT, all 40 sites of lesions in 23 patients were correctly located and diagnosed. The accuracy of SPECT/CT in detecting lesions was 100%, significantly higher than that of WBS (χ^2^ = 38.52, *P*<0.01). Of the 23 patients, 17 patients (73.9%, 18/23) with 31 lesion sites (77.5%, 31/40) presented with intratumoural calcification. Six patients had an absence of intratumoural calcification. Three of these patients had breast cancer, and one patient each had medullary thyroid cancer, endometrial cancer and liver metastatic disease from lung cancer.

## Discussion

^99m^Tc-MDP uptake in extraosseous neoplasms is occasionally encountered by WBS in clinical practice. However, to the best of our knowledge, no previous study has measured the incidence of such unusual uptake by WBS and SPECT/CT. In our series, ^99m^Tc-MDP uptake in extraosseous neoplasm was observed in 0.6% by WBS and 1.1% by SPECT/CT, usually localized in the breast, liver, and lung.

Radiological familiarity with ^99m^Tc-MDP uptake in extraosseous neoplasms is crucial to differentiate form osseous metastasis and prevent unnecessary treatment. However, the diagnosis of such extraosseous uptake can be a challenge if WBS is used alone, since extraosseous uptake frequently overlaps with skeletal structures, often mimicking osseous metastasis. Of the cases examined in our study, WBS had an accuracy of only 35% (14/40) based on the standard criteria. In our study [9, 10] and those of others [2, 16], SPECT/CT effectively resolved diagnostic uncertainty. In the present study, SPECT/CT correctly located and diagnosed all 40 sites (100%) of lesions in 23 patients. Early detection or exclusion of osseous metastasis has a profound influence on the management of patients with known malignancy. Therefore, the use of SPECT/CT is likely to result in altering patient management.

Nevertheless, the exact mechanism of ^99m^Tc-MDP uptake in extraosseous neoplasms is difficult to identify with precision, and various factors have been proposed. In some studies, histological evidence of calcium deposition has been proposed to be the most important factor [4, 6, 7, 12, 14, 15]. In our series, intratumoural calcification was noted in 31 lesion sites (77.5%, 31/40). We performed SPECT/CT in two patients with osteosarcoma in which the fused functional and anatomic images allowed increased sensitivity for the detection of lymph nodes, pleura, lung, subcutaneous and peritoneal metastasis due to metastatic lesions with calcification. Similar findings were reported by Mebarki M and colleagues [12] for the investigation of osteosarcoma. Classically, primary osteosarcoma and its metastases show extraosseous uptake due to bone matrix formation. Adenocarcinoma (both primary and metastatic) may calcify and accumulate ^99m^Tc-MDP, including carcinomas of the breast and lung, as well as colorectal cancer and ovarian cancer [14, 15, 17]. Among the primary malignancies in our series, breast cancer (*n* = 24) most frequently showed extraosseous uptake of ^99m^Tc-MDP. In our series, increased ^99m^Tc-MDP uptake was also described in a wide variety of primary neoplasms, including ovarian thecoma, malignant teratoma, and hepatocellular carcinoma. Extraosseous uptake in metastatic lesions were most frequently found in the liver, accounting for 42.3% (11/26) of lesions. The exact mechanism of extraosseous uptake of ^99m^Tc-MDP in breast, lung, and colorectal cancer is that adenocarcinoma and its metastases possess a mucinous component that may calcify because of internal tumour glycoprotein that binds calcium [14, 15, 17]. In addition, studies reported that calcification in colorectal liver metastasis is generally considered a good indicator of the response to treatment [18]. Therefore, WBS and SPECT/CT may be a potentially useful imaging modality to evaluate responses to treatment for patients with colorectal liver metastasis. Six patients had an absence of intratumoural calcification. Three of these patients had breast cancer, and one patient each had medullary thyroid cancer, endometrial cancer and liver metastatic disease from lung cancer. The mechanism of ^99m^Tc-MDP uptake in these patients may be increased tumour vascularity or hair-like calcification that is invisible on CT [14, 15].

This study has some limitations. First, the study was limited by its retrospective design, and the sample size was not large. Second, the actual rates of extraosseous uptake may have been underestimated because some lesions may be missed by WBS due to extraosseous ^99m^Tc-MDP uptake equal to or lower than that of adjacent bone; therefore, further SPECT/CT was not performed. Third, some lesions were not confirmed by histology, and the exact mechanism of extraosseous uptake may remain unknown in these patients.

## Conclusion

^99m^Tc-MDP uptake in extraosseous neoplasms can be observed as 0.6% on WBS and is usually localized to the breast, liver, and lung. Nuclear physicians should be familiar with such extraosseous uptake when interpreting WBSs. SPECT/CT offers better accuracy than WBS alone for locating the majority of lesions present with intratumoural calcification.

## Data Availability

The dataset supporting the conclusions of this article is included within the article. Data and materials during the current study are available from the corresponding author upon reasonable request.

## Abbreviations

^99m^Tc-MDP: ^99m^Tc-labeled methylene diphosphonate
SPECT/CT: Single photon emission computed tomography/computed tomography
WBS: Whole-body bone scans
